# Evaluation of Mineral Concentrations in Maternal Serum Before and After Birth and in Newborn Cord Blood Postpartum—Preliminary Study

**DOI:** 10.1007/s12011-017-1109-9

**Published:** 2017-08-01

**Authors:** Rafał Kocyłowski, Iwona Lewicka, Mariusz Grzesiak, Zuzanna Gaj, Przemysław Oszukowski, Constantin von Kaisenberg, Joanna Suliburska

**Affiliations:** 1 Department of Perinatology and Gynecology, Polish Mother’s Memorial Hospital Research Institute, ul. Rzgowska 281/289, 93-338 Łódź, Poland; 2 PreMediCare New Med Medical Centre, ul. Drużbickiego 13, 61-693 Poznań, Poland; 3 Department of Human Nutrition and Hygiene, Poznan University of Life Sciences, ul. Wojska Polskiego 31, 60-624 Poznań, Poland; 4 Department of Gynecology and Obstetrics, Polish Mother’s Memorial Hospital Research Institute, ul. Rzgowska 281/289, 93-338 Łódź, Poland; 5 Scientific Laboratory of the Center of Medical Laboratory Diagnostics and Screening, Polish Mother’s Memorial Hospital Research Institute, ul. Rzgowska 281/289, 93-338 Łódź, Poland; 6 Department of Obstetrics and Gynecology, Hannover Medical School, Carl-Neuberg-Str. 1, 30625 Hannover, Germany

**Keywords:** Minerals, Pregnancy, Delivery, Umbilical cord blood

## Abstract

The mineral levels in maternal serum change during pregnancy and may be correlated with those of newborn cord blood. The aim of this study was to evaluate the concentrations of calcium (Ca), magnesium (Mg), zinc (Zn), iron (Fe), and copper (Cu) in maternal blood before and after delivery and in umbilical cord vein and artery serum. The study was carried out in 64 Caucasian pregnant women who delivered in a district hospital in Greater Poland region, aged 28.1 ± 5.4 years, with a mean gestational age of 39.2 ± 1.3 weeks. Blood samples were taken from women 2–8 h before delivery and immediately after childbirth. The umbilical cord artery and vein blood of newborns was obtained immediately after childbirth. The levels of minerals in serum were determined by flame atomic absorption spectrometry. A significant drop in the concentrations of Mg (17.71 ± 1.51 vs 17.07 ± 1.61 μg/ml; *p* < 0.007), Fe (1.08 ± 0.46 vs 0.82 ± 0.35 μg/ml; *p* < 0.0004), and Zn (0.63 ± 0.17 vs 0.46 ± 0.16; *p* < 0.0001) in maternal serum was found after delivery. Moreover, higher levels of Ca, Fe, and Zn and lower levels of Cu were observed in the umbilical vein (Ca: 102.80 ± 7.80 μg/ml; *p* < 0.0001, Fe: 1.96 ± 0.43 μg/ml; *p* < 0.0001, Zn: 0.65 ± 0.16 μg/ml; *p* < 0.0001, Cu: 0.36 ± 0.09 μg/ml; *p* < 0.0001) and in the umbilical artery cord blood (Ca: 98.07 ± 8.18 μg/ml; *p* < 0.0001, Fe: 1.63 ± 0.30 μg/ml; *p* < 0.0001, Zn: 0.65 ± 0.15 μg/ml; *p* < 0.0001, and Cu: 0.36 ± 0.10 μg/ml; *p* < 0.0001) compared to the maternal serum (Ca: 85.05 ± 10.76 μg/ml, Fe: 0.82 ± 0.35 μg/ml, Zn: 0.46 ± 0.16 μg/ml, and Cu: 1.90 ± 0.35 μg/ml). Fe levels in the cord artery serum negatively correlated with blood loss during delivery (*R* = −0.48; *p* = 0.01), while the Ca concentration in the maternal serum after birth decreased with the age of the women (*R* = −0.25; *p* = 0.03). In conclusion, it seems that the process of birth alters the mineral levels in pregnant women’s blood. Moreover, it was found that blood loss and the age of the mother are associated with mineral concentrations in the maternal serum and cord artery blood.

## Introduction

There is a significant link between mineral status in pregnant women and fetal development, as well as with the subsequent health of the neonate. Animal experiments and clinical trials have demonstrated that a decrease in women’s mineral status during the preconception period and during pregnancy is associated with a higher risk of developing anemia, preeclampsia, and other obstetric complications [1, 2]. Mineral deficiencies are also considered to be contributing factors in premature birth, miscarriage, intrauterine growth restriction, birth defects, and immune system impairment. The available research suggests a causal relationship between the low intake of certain minerals during fetal life and the prevalence of chronic diseases later in adulthood [3–6].

In the early stages of pregnancy when the fetal skin is not yet keratinized, minerals are transported from the amniotic fluid to the fetus via simple diffusion. Later in pregnancy, the placenta and umbilical cord blood play an essential role in the transfer of minerals from the mother to the fetus; this transfer can be assessed by comparing mineral concentrations in maternal and cord serum [7]. There are several factors that may influence mineral status in pregnant women, such as food intake, supplementation, week of gestation, age, health status, and smoking status [3, 7]. It is suggested that stress and blood loss during delivery may affect mineral status in women; it also seems that the mineral composition in maternal serum may be correlated with that of newborn cord blood [1]. Taking this into account, the aim of this study was to assess the concentration of magnesium, zinc, iron, and copper levels in maternal blood before and after delivery, and also in cord serum (derived from the umbilical vein and artery separately).

## Materials and Methods

The study protocol was approved by the Bioethics Commission at Poznań University of Medical Sciences (approval no. 1800/04) and the Bioethics Commission at the Research Ethical Committee of the Polish Mother’s Memorial Hospital Research Institute (approval no. 50/2016). Informed consent was obtained from all women. The study was performed in accordance with the Helsinki Declaration.

## Study Population

The study was carried out in a district hospital and involved 64 Caucasian low-risk pregnant women from Greater Poland aged 28.1 ± 5.4. Average parity of women was 1.9 ± 1.2 and average gravida 2.0 ± 1.4. It was noticed that 45% women were in their second pregnancy and 41% of women had their second delivery. The mean gestational age was 39.2 ± 1.3 weeks. The study involved 35 (55%) male and 29 (45%) female babies. The average maternal bodyweight at birth was 75.5 ± 10.4 kg. The study was conducted between years 2012 and 2017.

The inclusion criteria were low-risk term singleton pregnancies, spontaneous vaginal delivery or Cesarean section, umbilical artery pH above 7.1, 5-min Apgar score above 7, neonatal birth weight within normal ranges (10th–90th centile), and pregnancy without complications (e.g., hypertension, preeclampsia, diabetes, obesity). The exclusion criteria were neonates and mothers with genetic defects, twin (or other multifetal) pregnancy, preterm birth below 34 weeks of pregnancy, fetal growth abnormalities (IUGR, macrosomia), and the supply of drugs affecting mineral and vitamin status.

Blood samples were taken from parturient women (those with regular uterine contractions) in the first stage of labor within 2–8 h before delivery and right after childbirth in the second stage of labor. The umbilical cord artery and vein blood of the newborns was obtained right after childbirth in the third stage of labor. The whole blood was then centrifuged and serum was stored at −80 °C. The loss of blood during delivery was determined by a semiquantitative method. A bedpan (kidney dish) method was chosen to measure blood loss during vaginal deliveries, and visual method for cesarean sections. Whenever blood clot was present, the estimation of blood loss changed by 30–50% depending on the proportion between liquid and clotted blood. Despite other methods of blood loss estimation seem superior like colorimetric photometry for cesarean births or mixed direct with gravimetric ones for vaginal deliveries, they appear less practical due to their costs and complexity.

The full characteristics of the subjects are presented in Table 1. All subjects were informed of the study’s aims, procedures, and measurement methods, and the individual consent of each patient was obtained.

**Table 1:** Anthropometric and demographic parameters of the study population (*n* = 64, mean ± SD)

Parameters	Values
Age (years)	28.1 ± 5.4
Maternal weight (kg)	75.5 ± 10.4
Parity	1.9 ± 1.2
Gravida	2.0 ± 1.4
Cesarean section (%)	14
Blood loss (ml)	298.4 ± 110.3
Gestational age	39.2 ± 1.3
Newborn gender (F/M)	29/35
Newborn birthweight (g)	3524.6 ± 534.8

*SD* standard deviation, *F* female, *M* male

## Measurement of Mineral Elements in Maternal and Newborn Blood Serum

The levels of calcium, magnesium, iron, zinc, and copper in the serum sample were determined by flame atomic absorption spectrometry (using a Zeiss AAS-3 spectrometer with deuterium background correction). In order to obtain the concentration of the plasma elements, the samples were diluted (*v*/*v* 1:1) as follows: for iron, zinc, and copper, 0.01% Triton X-100 (Merck) was used, while for the calcium and magnesium, aqueous solutions consisting of 0.01% Triton X-100 (Merck) and 0.05% lanthanum chloride (Merck) were used. The amounts of iron, zinc, copper, calcium, and magnesium in the plasma samples were determined at the following wavelengths, respectively: 248.3, 213.9, 324.8, 422.7, and 285.2 nm. The accuracy of the method was verified using certified reference material (Hum Asy Control 2, Randox) and was 95, 99, 94, 99, and 101% for calcium, magnesium, iron, zinc, and copper, respectively.

## Statistical Analysis

A detailed statistical analysis was performed using Statistica 10 for Windows. The normality of the variables’ distribution was verified using the Shapiro–Wilk test. The Wilcoxon Signed Rank Test was used to compare differences between groups for all the studied parameters. Simple associations between parameters were calculated as the Spearman coefficient of correlation. The level of statistical significance was set to *p* < 0.05.

## Results

The mean and median concentrations of minerals are shown in Table 2. It was found that the concentrations of magnesium, iron, and zinc in maternal serum significantly decreased after childbirth. Calcium, iron, and zinc levels in umbilical cord serum were markedly higher than in maternal serum after birth. In contrast, the concentration of copper in the umbilical cord serum was significantly lower than in the mother’s serum. Moreover, a markedly lower calcium, magnesium, and iron level was observed in the umbilical cord artery than in the vein.

**Table 2:** Concentration of mineral elements in maternal serum before and after birth and in umbilical artery and vein cord blood

Parameter (μg/ml)	Maternal serum		Umbilical cord serum	
	Before birth	After birth	Vein	Artery
Ca				
Mean ± SD Median	84.84 ± 7.79 84.2	85.05 ± 10.76 85.36	102.80 ± 7.80 101.98^a^	98.07 ± 8.18 97.58^a^
*p*	NS		< 0.0001	
Mg				
Mean ± SD Median	17.71 ± 1.51 17.92^a^	17.07 ± 1.61 17.24	17.68 ± 1.60 17.84	17.40 ± 1.39 17.34
*p*	0.007		0.04	
Fe				
Mean ± SD Median	1.08 ± 0.46 1.04^a^	0.82 ± 0.35 0.74	1.96 ± 0.43 2.03^a^	1.63 ± 0.30 1.61^a^
*p*	0.0004		0.003	
Zn				
Mean ± SD Median	0.63 ± 0.17 0.64^a^	0.46 ± 0.16 0.46	0.65 ± 0.16 0.63^a^	0.65 ± 0.15 0.64^a^
*p*	< 0.0001		NS	
Cu				
Mean ± SD Median	1.91 ± 0.40 1.91	1.90 ± 0.35 1.87	0.36 ± 0.09 0.36^a^	0.36 ± 0.10 0.36^a^
*p*	NS		NS	

*SD* standard deviation, *NS* not significant

^a^Significant differences vs after birth (*p* < 0.0001)

**Fig. 1:**
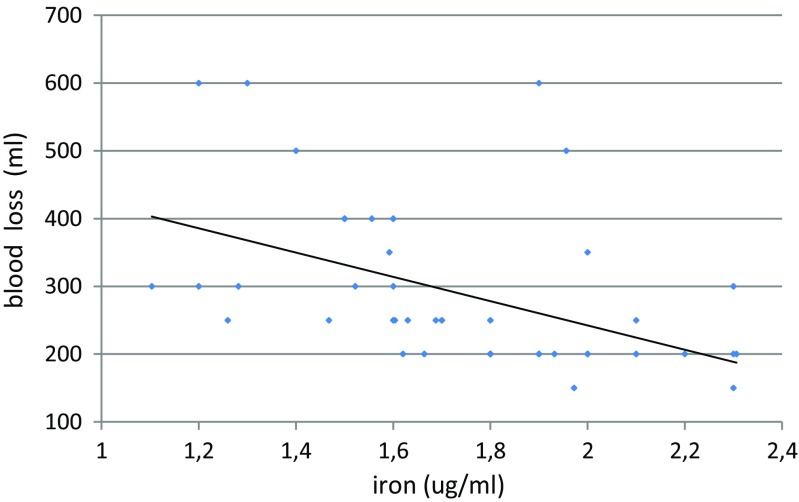
Correlation between blood loss and iron concentration in artery cord blood serum (*R* = −0.48; *p* = 0.01)

**Fig. 2:**
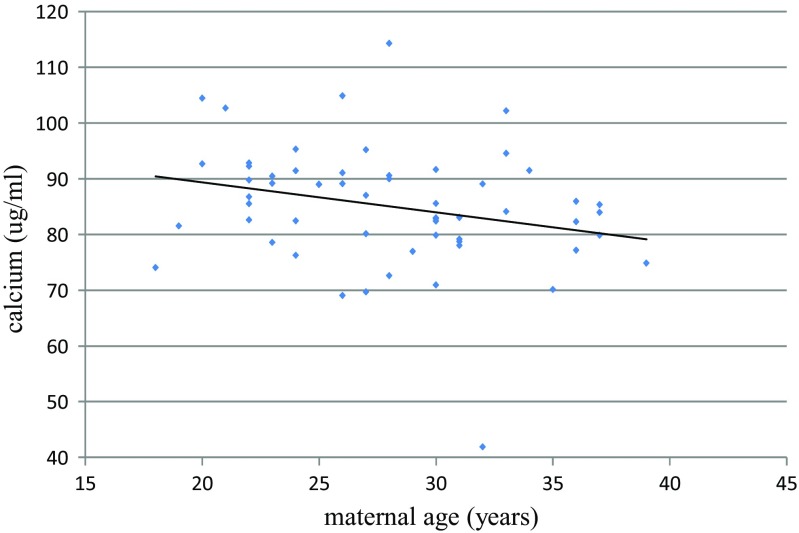
Correlation between maternal age and calcium concentration in maternal serum after childbirth (*R* = −0.29; *p* = 0.03)

Table 3 shows significant correlations between mineral concentration and selected parameters. Maternal blood loss during delivery correlated negatively with the concentrations of the investigated mineral elements (except magnesium) in umbilical cord artery serum, and a strong correlation (*R* = −0.48) was observed between iron and blood loss (Fig. 1). Maternal age negatively correlated with calcium levels in the mother’s serum after childbirth (Fig. 2). Moreover, positive correlations were observed in maternal serum between calcium and magnesium and between zinc and copper after delivery. A correlation was found between the level of mineral elements other than iron in the cord vein and artery blood. Marked relations between magnesium and calcium, and also between iron and zinc and copper, were observed in cord blood.

**Table 3:** Significant correlation between parameters

Blood loss	Ca2	Zn2	CaV
CaA *R* = −0.25; *p* = 0.03 FeA *R* = −0.48; *p* = 0.01 ZnA *R* = −0.21; *p* = 0.04 CuA *R* = −0.25; *p* = 0.04	Age *R* = −0.29; *p* = 0.03 Mg2 *R* = 0.41; *p* = 0.02	Cu2 *R* = 0.29; *p* = 0.03	CaA *R* = 0.51; *p* = 0.01 MgV *R* = 0.47; *p* = 0.02
MgV	FeV	ZnV	CuV
MgA *R* = 0.43; *p* = 0.03 CaV *R* = 0.51; *p* = 0.02	ZnA *R* = 0.37; *p* = 0.03 CuV *R* = −0.34; *p* = 0.03 CuA *R* = −0.49; *p* = 0.02	ZnA *R* = 0.45; *p* = 0.01 FeA *R* = 0.63; *p* = 0.01	CuA *R* = 0.41; *p* = 0.02

Ca2; Mg2; Zn2; Cu2—concentration of minerals after birth

CaA; MgA; FeA; ZnA; CuA—concentration of minerals in artery cord blood

CaV; MgV; FeV; ZnV; CuV—concentration of minerals in vein cord blood

## Discussion

In this study, a significant decrease was observed in the concentrations of magnesium, iron, and zinc in maternal serum after delivery. Moreover, we found higher levels of calcium, iron, and zinc and also lower levels of copper in umbilical cord blood than in maternal serum.

The maternal average serum calcium concentration before delivery, as determined in the present study, is similar to the values reported by other authors [2, 8, 9]. In several studies, a decrease in maternal calcium concentration has been observed as pregnancy progressed [8, 10, 11]. In both this and previous studies, it was observed that maternal calcium concentration was slightly lower than in cord blood [8, 10, 12]. This insignificant difference between serum calcium concentration of the mother and fetus can be explained by the metabolic changes in the female body during pregnancy, which are designed to compensate for the increased maternal and fetal demand for calcium. Pregnancy is associated with a significant increase in serum concentration of estrogen and progesterone in the female body, which subsequently affects the concentration of many substances, including calcium. A decrease in the urinary excretion of calcium and intense bone remineralization are observed during pregnancy [2]. Additionally, an increase in the synthesis of 1,25-dihydroxyvitamin D results in an increase in the intestinal absorption of calcium and its storage in the mother’s skeleton, to supply the fetus with adequate calcium later in pregnancy [2]. Age of pregnant women may influence the mineral status, especially calcium level. In women who give birth after age 38, increased risk of hip fracture and osteoporosis related with low calcium status was observed [13].

The serum magnesium concentration of the maternal and cord blood determined in our study was lower than in the studies of other authors [2, 8, 14]. The slight differences in these values may be the result of different methods of biochemical analysis of the blood serum or of differences in the populations of pregnant women studied. Tabrizi and Pakdel [2] and Khoushabi et al. [8] reported no statistically significant changes in magnesium concentration in the three trimesters of pregnancy. In contrary, Kozielec et al. [15] observed that the concentration of magnesium in cord serum was significantly higher than in maternal serum and that the two were positively correlated. Moreover, a positive correlation was found between magnesium levels in the cord serum and in the hair of newborns. Kozielec et al. [15] also observed that fetal gender affected cord serum magnesium concentration, with male fetuses having a higher concentration of magnesium in their cord blood than female fetuses. Our analysis showed a statistically significant difference in the maternal serum magnesium concentration before delivery, compared to magnesium levels after childbirth. The maternal serum concentration of magnesium after delivery was significantly lower, although no negative correlation was observed between magnesium concentration and blood loss during childbirth. Decreasing of magnesium in serum after delivery may be related to biochemical and hormonal changes in the maternal body during delivery and postpartum and also combined with intense stress and physical effort. Lower albumin and higher cortisol levels were found in serum after delivery [16, 17]. It is known that much of magnesium in the blood is carried by albumin, and also, a relation between emotional and physical stress and loss of magnesium was shown in other experimental and human studies [16, 18]. Lower magnesium concentration in the maternal blood after delivery may increase the risk of postpartum anxiety and depression which are prevalent disorders in women [19].

This study has shown a significant relationship between the concentration of magnesium and calcium in maternal and umbilical cord blood serum. Magnesium and calcium concentrations in venous cord blood showed a significant positive correlation. Similarly, a positive relationship was observed in both of these elements in the maternal serum after delivery.

A significant difference was observed in our study in maternal iron levels before and after delivery. The concentration of this element in maternal serum after childbirth was significantly lower than prior to delivery. In several human studies, a decrease has been observed in the serum concentration of iron as the pregnancy progresses [2, 8, 11, 20, 21]. In the present study, a significantly higher concentration of iron in cord blood serum (both arterial and venous) was observed than in the maternal blood serum obtained after delivery. These results are consistent with the observations of Jariwala et al. [1], who also found significantly higher iron concentrations in venous umbilical cord serum than in the arterial serum. These observations may reflect the fact that, as the fetus develops, the mother’s iron stores reduce. The highest demand for iron occurs in the third trimester of pregnancy, when the fetal iron requirement and storage is greatest. The 1st year of a child’s life is a time of rapid growth and development, and during the first 4–6 months of life, the child draws on iron stores accumulated during the third trimester of pregnancy [22].

This study has revealed a relationship between the concentrations of iron, copper, and zinc. A positive correlation was observed between zinc levels in venous umbilical cord blood serum and iron levels in serum derived from arterial umbilical cord blood. There is also a statistically significant positive correlation between serum iron concentration in venous cord blood and serum zinc concentration in arterial umbilical cord blood. Jariwala et al. [1], in their study of a population of Indian pregnant women, also noted a positive relationship between serum iron and zinc levels in umbilical cord blood. A similar relationship was noted in relation to the maternal serum concentration of iron and zinc. A negative correlation was reported with respect to the serum copper concentration of the arterial and venous umbilical cord blood.

Similarly to our results, Awadallah et al. [23] found higher concentrations of Fe and Zn and a lower level of Cu in cord blood than in maternal serum; however, they noticed a lower value of concentration for Fe and a higher for Zn and Cu compared to values in this study.

The maternal serum zinc concentrations observed in our study prior to delivery are similar to the results obtained by Jariwala et al. [1]. However, other studies have reported lower [21, 24] and higher serum concentrations of maternal zinc [25, 26]. The post-delivery maternal concentration of zinc identified in our study was significantly lower than its value before childbirth. This difference may be related to the negative correlation observed between blood loss during delivery and the concentration of certain minerals in the blood. We did not observe significant differences in the concentration of zinc in maternal serum before delivery or in arterial or venous umbilical cord blood. A statistically significant difference in zinc concentration was observed for maternal blood after delivery and for cord blood. Other authors have also noted significantly higher concentrations of zinc in umbilical cord blood than in maternal serum or colostrum [1, 8, 27]. This finding demonstrates the passive transport of zinc from mother to fetus through the placenta, which may result in an increase in zinc concentration in umbilical cord blood [25]. Other studies have highlighted that the distribution of zinc in maternal and cord blood shows a trend similar to the concentrations of iron [1, 8]. Our study, however, did not confirm these observations. Youssof et al. [28] found that maternal age and parity may influence Zn concentration in cord blood. Authors suggested that multiparous older mothers would require more Zn supplement during pregnancy. Decreasing Zn concentration with age and parity may be possible associated with catecholamine level in maternal and cord blood which affects Zn status [28]. In another study, a significant positive correlation between Zn level in cord blood and birth weight was observed [23]. In this study, we did not show a correlation between cord blood Zn concentration and maternal and neonatal parameters.

According to the literature, serum zinc concentrations during pregnancy are significantly lower than zinc concentrations in nonpregnant women [29]. It is suggested that this is due to a significant demand for zinc by the fetus, the development of the placenta, and the increase in blood volume during pregnancy [1]. No significant difference was observed between copper levels in maternal serum before and after delivery. Data from other studies has shown lower copper concentrations in maternal blood than we report here [1, 2]. Several studies have shown that the concentration of copper in pregnant women was significantly higher than in nonpregnant women [25, 26]. It is suggested that this is related to the increase in blood estrogen levels, which mobilize copper stores from tissues. In the present study, the serum copper concentrations of umbilical arteries and veins were similar. Furthermore, we found the copper concentrations in maternal serum to be much higher than in cord blood. These observations are consistent with the results obtained by other authors [1, 27]. However, Upadhyaya et al. [21] and Schulpis et al. [29] reported higher values of copper level in cord serum than we did. Iqbal et al. [27] observed significant inverse correlation between maternal age and copper level in maternal serum and cord blood but in this study, we did not confirm that association. However, we found a negative correlation between iron and copper concentrations in cord blood. These observations are not reflected in previously conducted studies and require further research.

Our study has some limitations. The study group should be larger to allow observation of the changes that occur in subgroups. We involved in this study only healthy women without complications. Moreover, we did not analyze the dietary intake, nutritional status of pregnant women, and supplement intake during pregnancy which may affect mineral concentrations in maternal and cord blood. Further investigation is needed to include all necessary factors and parameters to confirm and more explain obtained results.

## Conclusions

This study draws attention to the lack of significant correlation between the concentration of minerals in the blood serum of pregnant women and umbilical cord blood serum. Our results suggest that the process of birth, and also the age of the mother, changes mineral status in women just after delivery. Moreover, it was found that blood loss is associated with alteration of mineral concentrations in cord artery blood at birth.
